# Supplementary material for: Genomic and Molecular Identification of Genes Contributing to the Caspofungin Paradoxical Effect in Aspergillus fumigatus
Source: Microbiol Spectr. 2022 Sep 12;10(5):e00519-22. doi: 10.1128/spectrum.00519-22 (PMC9603777; doi:10.1128/spectrum.00519-22)
Supplement: Supplemental file 1 — Supplemental material. Download spectrum.00519-22-s0001.pdf, PDF file, 3.7 MB [file spectrum.00519-22-s0001.pdf]

Table S1. Growth rate (colony diameter in mm) and caspofungin paradoxical effect (Recovery Rate) quantification of 67 *A. fumgiatus* isolates in the presence of 0, 0.125, 0.25, 0.5, 1, and 8 ug/ml caspofungin.

Isolate	Minimal media r1	Minimal media r2	Caspo 0.125ug/ml r1	Caspo 0.125ug/ml r2	Caspo 0.250ug/ml r1	Caspo 0.250ug/ml r2	Caspo 0.500ug/ml r1	Caspo 0.500ug/ml r2	Caspo 1ug/ml r1	Caspo 1ug/ml r2	Capo 8ug/ml r1	Capo 8ug/ml r2	Recovery Rate	Source of Strain	SRA Accession Number
17993925	6.5	6.4	3	3.2	2.4	2.5	1.5	1.6	1	1	3.8	3.7	0.504587156	Current study	SRR16287627
200-89320	6.2	6.3	3.2	3.1	2.1	2.3	1.2	1.1	1	1	3.8	3.9	0.542857143	Current study	SRR16287628
A1163	6.8	6.7	3.5	3.5	2.6	2.8	1.7	1.7	1.1	1.2	3.5	3.5	0.419642857	Garcia-Rubio et al., 2018	SRR068950
Af293	6	6.1	3.3	3.2	1.4	1.4	1	1.1	0.7	0.8	3.8	4	0.594339623	http://www.westerdijkinstitute.nl/	SRR068952
AF72	3.2	3	1	1	1	0.9	0.5	0.6	0.5	0.5	0.8	0.9	0.134615385	Mosquera et al., 2002	SRR617721
Afs35	6.5	6.5	2.8	2.8	2	2.1	1.4	1.5	1.2	1.1	3.7	3.5	0.457943925	Kang et al., 2020	DRR146814
Afu-AF10	8	7.9	3	3.1	2	1.8	1.3	1.2	1	0.9	1	1	0.007142857	Chiba University, unpublished	SRR334209
ATCC204305	8	8	3	3.1	2.3	2.3	1.5	1.4	1	1	2.7	2.7	0.242857143	Garcia-Rubio et al., 2018	SRR7418943
ATCC46645	8	8	3.2	3.1	2	2.1	1.5	1.5	1.5	1.5	4	4	0.384615385	Garcia-Rubio et al., 2018	SRR7418935
CEA10	8.5	8.5	3	2.9	1.6	1.6	1.6	1.6	1.6	1.5	4.4	4.5	0.417266187	Fedorova et al., 2008	SRR7418934
CM2141	8	8	4	3.9	2.7	2.7	1.9	1.8	1.1	1.1	4	4.1	0.427536232	Garcia-Rubio et al., 2018	SRR7418947
CM237	8	8.1	4	4	2.5	2.5	1.7	1.6	1.5	1.4	3	3	0.234848485	Garcia-Rubio et al., 2018	SRR7418942
CM3248	7.5	7.6	3	3	1.9	1.9	1.3	1.4	1	1	1	1	0	Garcia-Rubio et al., 2018	SRR7418945
CM5419	7.5	7.6	4	3.9	3	3	2.1	2	1.5	1.6	4.2	4.3	0.45	Garcia-Rubio et al., 2018	SRR7418944
CM5757	8	8	3.8	3.8	3.1	3	2	2	2.5	2.4	4	4	0.333333333	Garcia-Rubio et al., 2018	SRR7418949
CM6126	8	8	4	4	3.5	3.5	2.5	2.6	1.5	1.6	4.3	4.2	0.418604651	Garcia-Rubio et al., 2018	SRR7418937
CM6458	8	7.9	5	4.9	4.3	4.2	3	3.1	1.5	1.5	4.5	4.5	0.465116279	Garcia-Rubio et al., 2018	SRR7418936
CM7510	8	8	3.3	3.1	2.8	2.9	1.7	1.7	1.1	1	3.8	3.9	0.402877698	Garcia-Rubio et al., 2018	SRR7418939
CM7555	8	7.9	5.1	5.1	4.8	4.7	3.4	3.5	2.6	2.5	4.7	4.5	0.37962963	Garcia-Rubio et al., 2018	SRR7418938
CNM-CM2495	7.5	7.5	3.2	3.1	1.5	1.5	1	1	1	1	1	1	0	Garcia-Rubio et al., 2018	SRR7418930
CNM-CM2730	8	7.9	2.8	2.8	1	1	1	1	1	1	1	1	0	Garcia-Rubio et al., 2018	SRR7418924
CNM-CM2733	7.5	7.5	3.2	3.2	1.3	1.4	1	1	1	1	1	1	0	Garcia-Rubio et al., 2018	SRR7418923
CNM-CM3249	7.2	7.2	2.7	2.7	1.5	1.5	1	1	1	1	1	0.9	0	Garcia-Rubio et al., 2018	SRR7418926

CNM-CM3262	7.5	7.5	3.7	3.6	2.5	2.5	1.6	1.5	1.2	1.1	4.5	4.5	0.527559055	Garcia-Rubio et al., 2018	SRR7418922
CNM-CM3720	6.4	6.5	1.8	1.8	1.3	1.3	1	1	1	1	2.5	2.8	0.302752294	Garcia-Rubio et al., 2018	SRR7418927
CNM-CM4602	7.8	7.9	2.6	2.5	1.5	1.5	1	1	0.7	0.8	1	1	0.035211268	Garcia-Rubio et al., 2018	SRR7418928
CNM-CM4946	7.5	7.57	2.7	2.6	1.5	1.6	0.9	0.8	0.8	0.9	0.9	0.9	0.007479432	Garcia-Rubio et al., 2018	SRR7418948
CNM-CM7560	7.5	7.5	3.7	3.7	2.8	2.9	1.8	1.9	1	0.9	1	1	0.007633588	Garcia-Rubio et al., 2018	SRR7418925
CNM-CM7632	7.8	7.7	3	3.1	2.1	2	1.4	1.5	1	1	1	1	0	Garcia-Rubio et al., 2018	SRR7418941
CNM-CM8057	6.8	6.7	3	3.1	0.6	0.6	1.5	1.6	1.2	1.1	4	4	0.552845528	Garcia-Rubio et al., 2018	SRR10592633
CNM-CM8686	7	7.2	3	3.1	1.7	1.7	1.2	1.1	1.2	1.1	4.2	4.1	0.504201681	Garcia-Rubio et al., 2018	SRR10592630
CNM-CM8689	7	7	3.2	3.2	2.2	2.1	1.2	1.2	1	1	3.2	3.1	0.358333333	Garcia-Rubio et al., 2018	SRR10592629
CNM-CM8714	7.5	7.6	3.3	3.4	2	2.1	1.3	1.3	1.3	1.4	4.1	1.1	0.208	Garcia-Rubio et al., 2018	SRR10592632
CNM-CM8812	7	7.1	3	3	1.3	1.2	1.1	1	0.9	1	1	1	0.008196721	Garcia-Rubio et al., 2018	SRR10592631
F12041	6.5	6.2	2.5	2.6	2.3	2.2	1.3	1.2	1	1	3.3	3	0.401869159	Bromley et al., 2017	SRR617723
F12636	6	6	2.6	2.5	2.3	2.1	1.3	1.3	1	1	3.1	3	0.41	Bromley et al., 2017	SRR617725
F13535	7	7	2.3	2.5	2	2.1	1.5	1.4	1	0.9	2.5	2.5	0.256198347	Bromley et al., 2017	SRR617726
F13952	5.2	5.1	2	2	1.3	1.3	1.2	1.1	1	1	2.5	2.4	0.34939759	Bromley et al., 2017	SRR617728
F14946	6.5	6.5	2.5	2.5	1.5	1.4	1	1	1	0.9	2.6	2.5	0.288288288	Bromley et al., 2017	SRR159252
F17764	7	7.1	2.5	2.5	1.9	1.9	1.2	1	1	1	2.5	2.5	0.247933884	Balajee et al., 2007	SRR617745
F7763	7	6.8	2.5	2.5	2.1	2.1	1.5	1.3	1	1	1	1	0	Lepak et al., 2013	SRR617744
IF1SW-F4	6.6	6.5	3.5	3.4	2.1	1.9	1	1	0.9	0.9	3.9	3.8	0.522123894	Knox et a., 2016	SRR4002444
IFM55369	6.2	6.2	2.5	2.3	1.2	1	0.9	0.8	1	1	1	1	0.028037383	Hagiwara et al., 2014	DRX013572
IFM58026	5	5.1	3.7	3.8	2	2.1	1.5	1.5	1.6	1.5	1.4	1.5	0	Hagiwara et al., 2014	DRX015829
IFM58029	6.2	6	4.5	4.3	1.9	1.9	1.7	1.6	1.4	1.6	1	1	0	Hagiwara et al., 2014	DRX015830
IFM59056	7	7	4.8	4.7	3.5	3.6	1.5	1.7	1	1	2	2.1	0.175	Hagiwara et al., 2014	DRX013573
IFM59073	7.2	7.2	3.7	3.5	2	2.1	1.9	1.8	0.9	1	1	1	0.008	Hagiwara et al., 2014	DRX013577
IFM59359	4.3	4.2	2.6	2.6	1.5	1.5	1.3	1.2	1.5	1.6	2	2	0.25	Hagiwara et al., 2014	DRX013574
IFM59361	4	4.1	0.4	0.4	0.5	0.4	0.5	0.6	0.6	0.5	0.9	0.8	0.123287671	Hagiwara et al., 2014	DRX013575
IFM59365	7	7.1	4.2	4.1	3	3	1.7	1.6	1	1	2.6	2.8	0.280991736	Hagiwara et al., 2014	DRX015832
IFM59777	7	7	5.3	5.4	3	3.1	1.5	1.4	1.4	1.5	1	1	0	Hagiwara et al., 2014	DRX015833
IFM60514	2	2.1	1.8	1.8	1.6	1.7	1.6	1.6	1.8	1.7	2.5	2.4	1.888888889	Hagiwara et al., 2014	DRX013576
IFM61118	7.5	7.5	3.4	3.5	1.9	2	1.1	1	1	1.1	1	0.9	0	Hagiwara et al., 2014	DRX015834
IFM61407	6.5	6.5	3.5	3.7	1.2	1.1	1	1.1	0.9	1	0.8	0.7	0	Hagiwara et al., 2014	DRX013578
IFM61578	6.2	6.2	2.1	2	1.3	1.4	1	1	1	1	0.9	1	0	Hagiwara et al., 2014	DRX015835

IFM61610	4.1	4.1	3.5	3.6	2.3	2.4	2	2.1	1.7	1.8	3.5	3.5	0.744680851	Hagiwara et al., 2014	DRX013579
IFM62516	5	5.1	3.3	3.2	2.5	2.5	1.6	1.6	1.4	1.5	2	1.9	0.138888889	Hagiwara et al., 2014	DRX015837
ISSFT-021	6.5	6.5	3	3.1	2.3	2.5	1	1.1	1	1	3.6	3.7	0.481818182	Knox et a., 2016	SRR4002443
MO54056	7.4	7.3	5.1	5	2.4	2.3	1.2	1.1	1	1	1	1.1	0.007874016	Lind et al., 2017	SRR5676587
MO68507	7.5	7.5	3.8	3.7	2	2	1.2	1	1	1	1	0.9	0	Lind et al., 2017	SRR5676586
MO69250	7.5	7.6	4	4	2.8	2.7	1.5	1.5	0.9	1	1.3	1.5	0.068181818	Lind et al., 2017	SRR5676589
MO78722	7.5	7.4	4.6	4.7	2.5	2.6	1.6	1.5	1.2	1.2	3.8	3.7	0.408	Lind et al., 2017	SRR5676591
MO79587	6.5	6.5	4.5	4.4	1.6	1.5	1	1	1	0.9	2.5	2.5	0.279279279	Lind et al., 2017	SRR5676590
MO89263	7.5	7.4	4	4.1	2.5	2.6	1	1	1	1	1	1	0	Lind et al., 2017	SRR5676593
MO91298	7	7.1	3.3	3	1.9	2	1.1	1.2	1	1	1.1	1	0.008264463	Lind et al., 2017	SRR5676592
TP-12	7.5	7.5	3	2.9	2.1	2	1	1	0.9	0.9	3.2	3.1	0.340909091	Garcia-Rubio et al., 2018	SRR7418940
TP32	7.8	7.9	2.1	2	1.7	1.8	1	1	1	1	1	1	0	Garcia-Rubio et al., 2018	SRR7418946

*MM = minimal media, Caspo = caspofungin, growth values = colony diameter (mm)

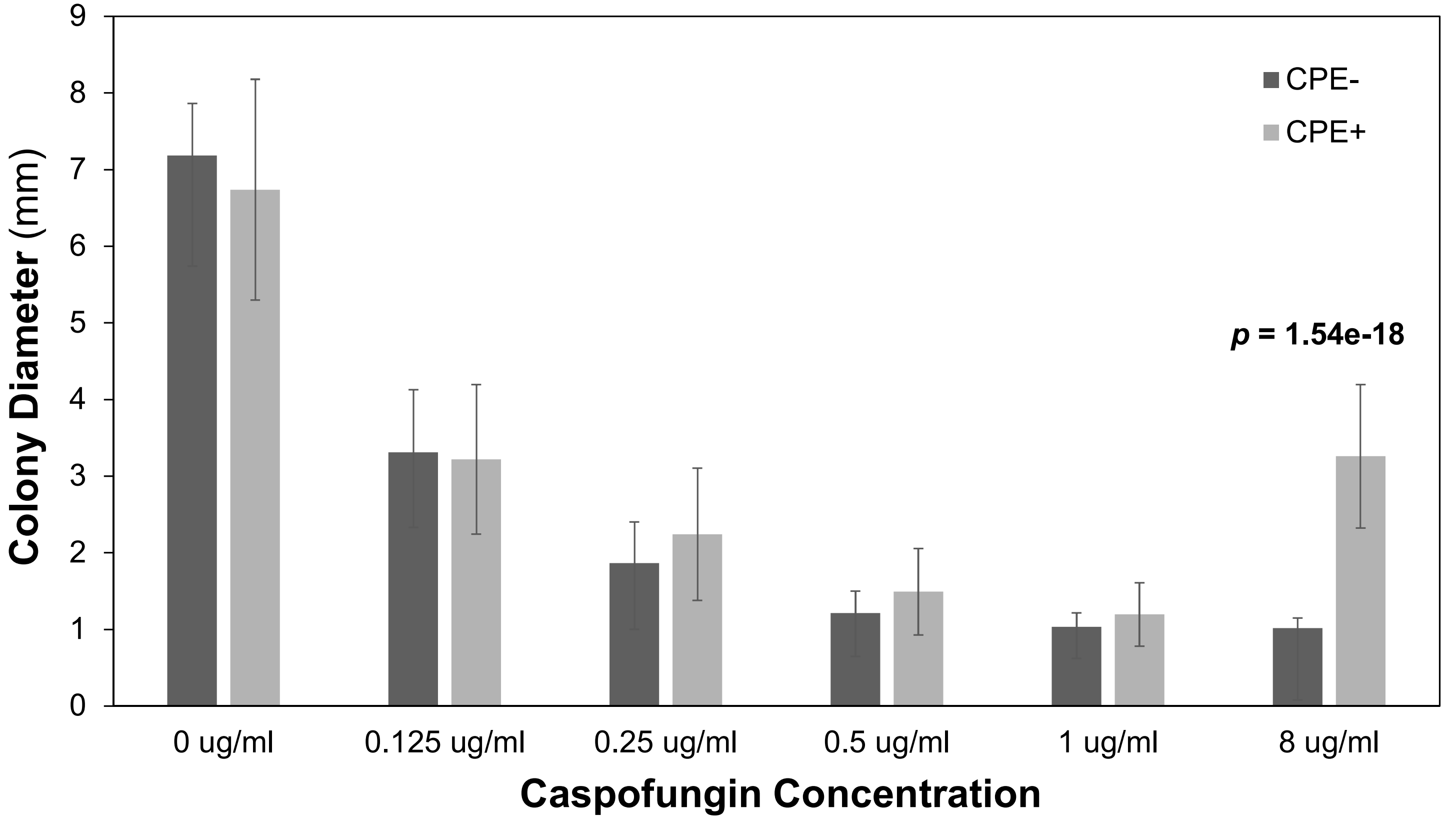

Figure S1. Growth of CPE+ (light gray) and CPE- (dark gray) isolates at varying concentrations of caspofungin. Growth differences between CPE- and CPE+ isolates only significantly differed at 8 ug/ml, where CPE+ grew faster. All other p-values > 0.05. Error bars represent standard deviation. N = 41 CPE+ and N = 26 CPE-.

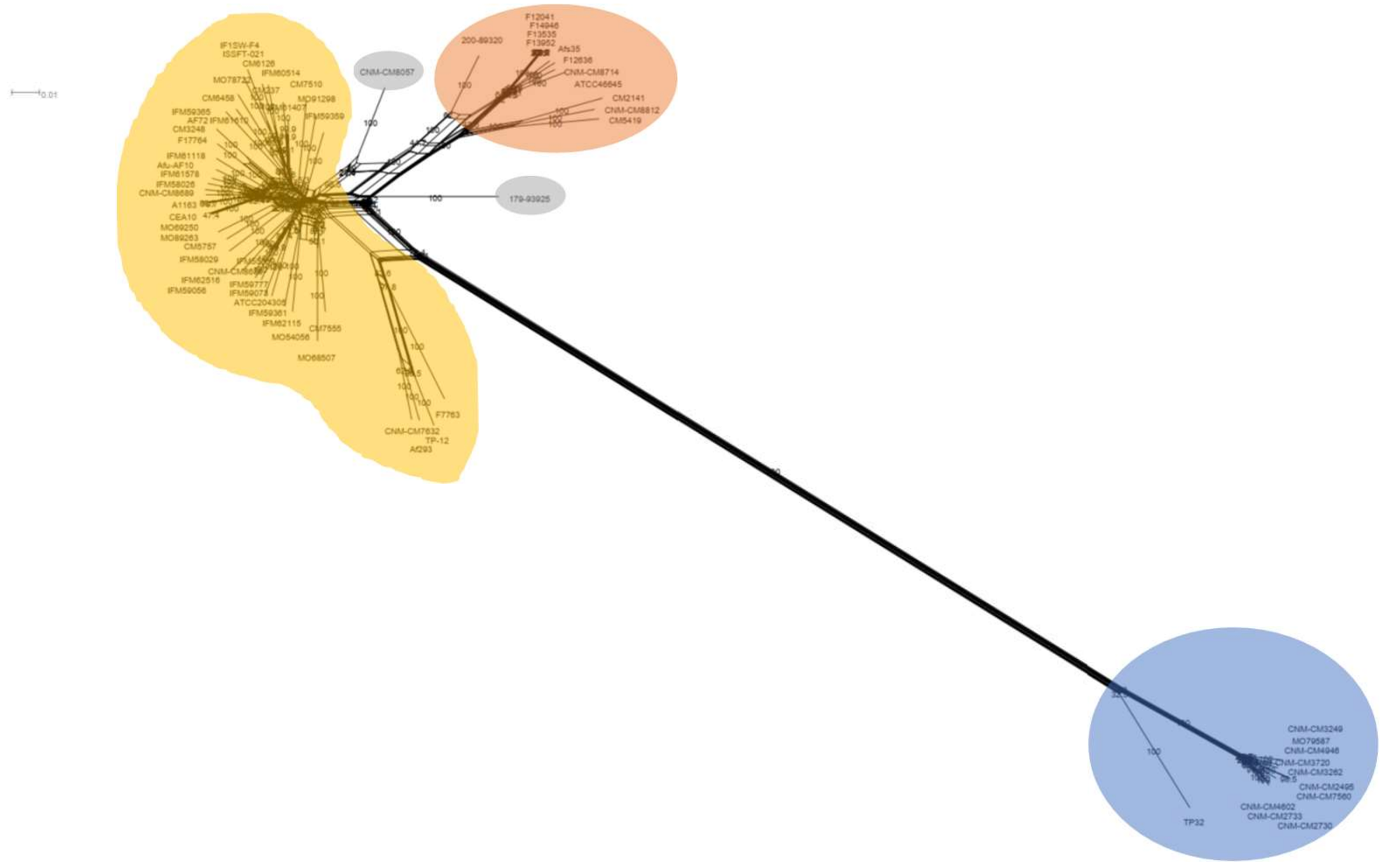

Figure S2. Phylogenetic network of the 67 *A. fumigatus* isolates inferred from 6,492 SNPs distributed across the genome. The neighbornet method was used to construct the network with 1,000 bootstrap replicates. Colors represent the major populations identified through PCA analysis (Figure 2).

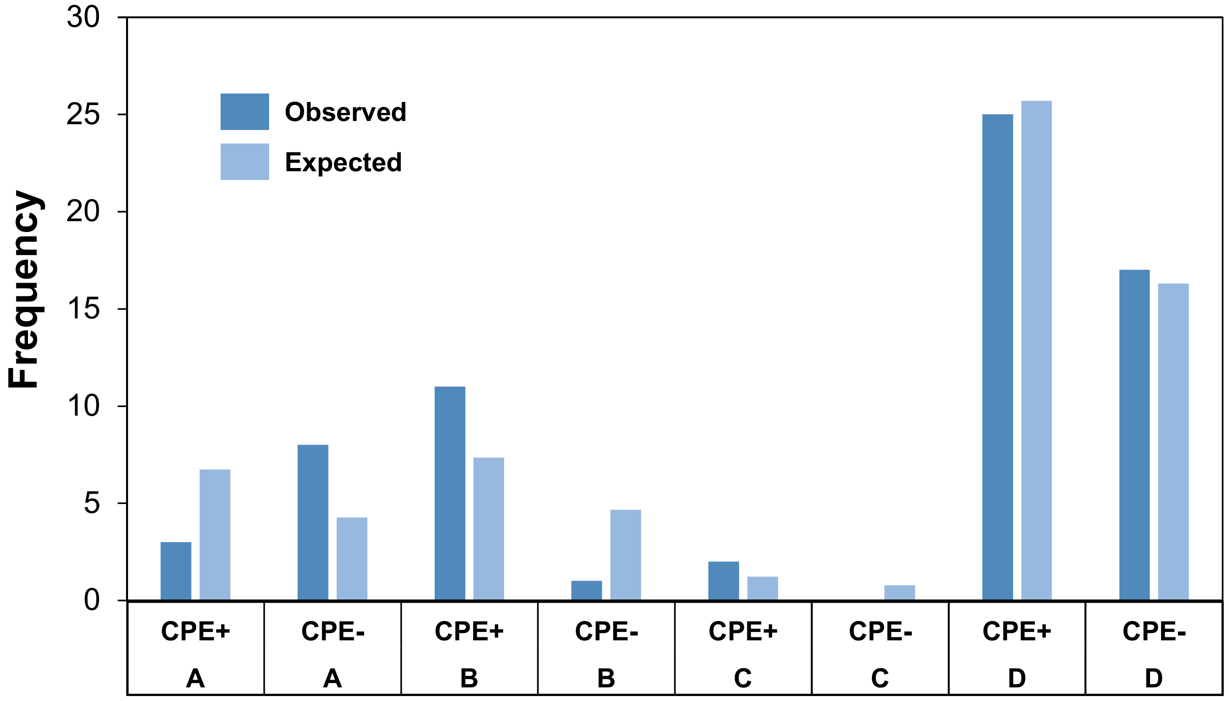

Figure S3. Observed versus expected frequencies of CPE+ and CPE- isolates across populations A-D assuming a random distribution. Dark blue and light blue represent observed and expected frequencies. A-D represent the 4 *A. fumigatus* populations.

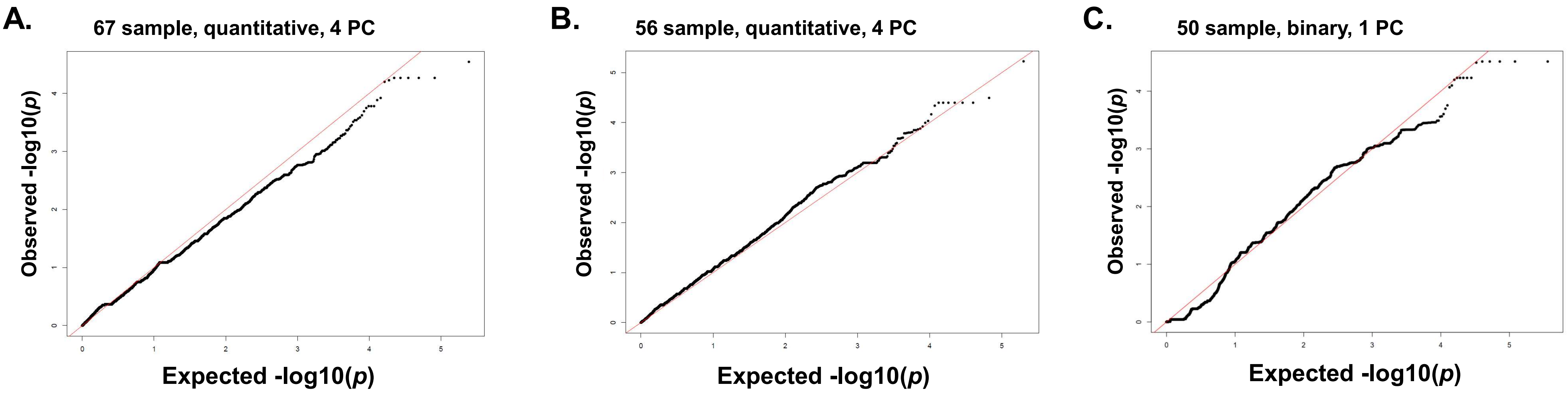

Figure S4. Quantile-Quantile plots of observed (Y-axis) vs. expected (X-axis) *p-values* in the (A) 67-sample, (B) 56-sample, and (C) 50-sample GWA analyses.

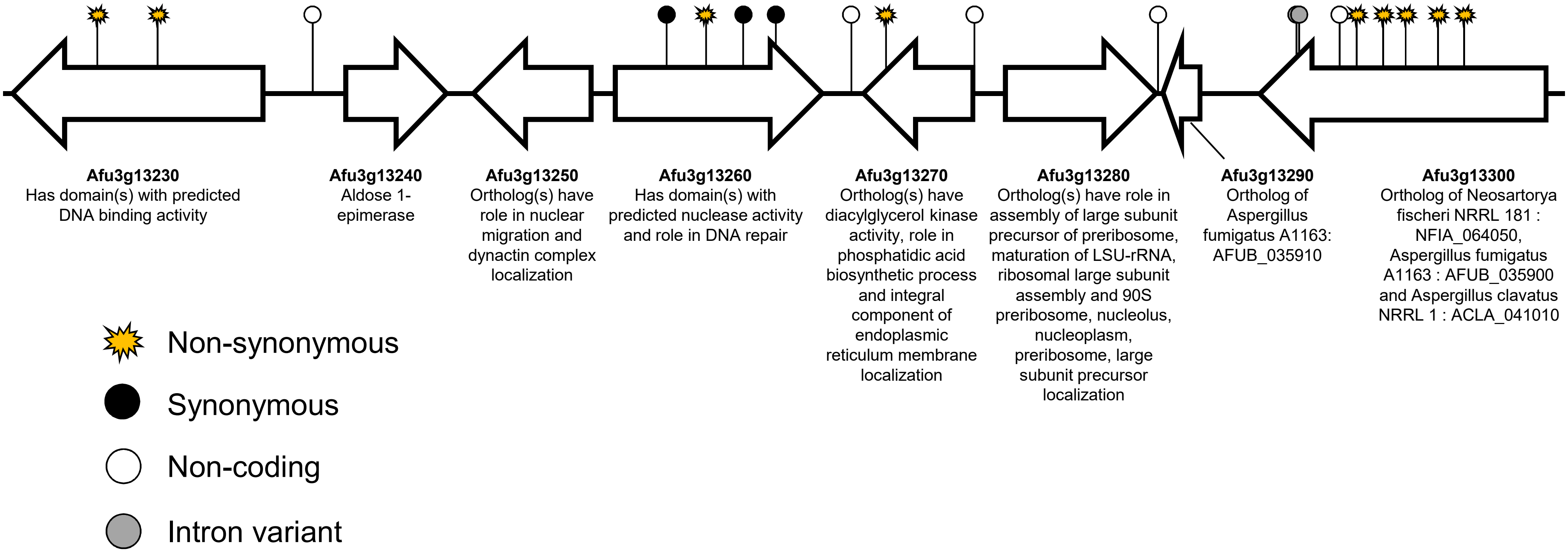

Figure S5. Schematic of chromosome 3 region containing several SNPs significantly associated with CPE. Arrows represent genes, with direction indication the direction of transcription. Lines with symbols represent the location and type of SNPs associated with CPE. Gene symbol and functional descriptions (as provided by FungiDB) are provided below each gene.

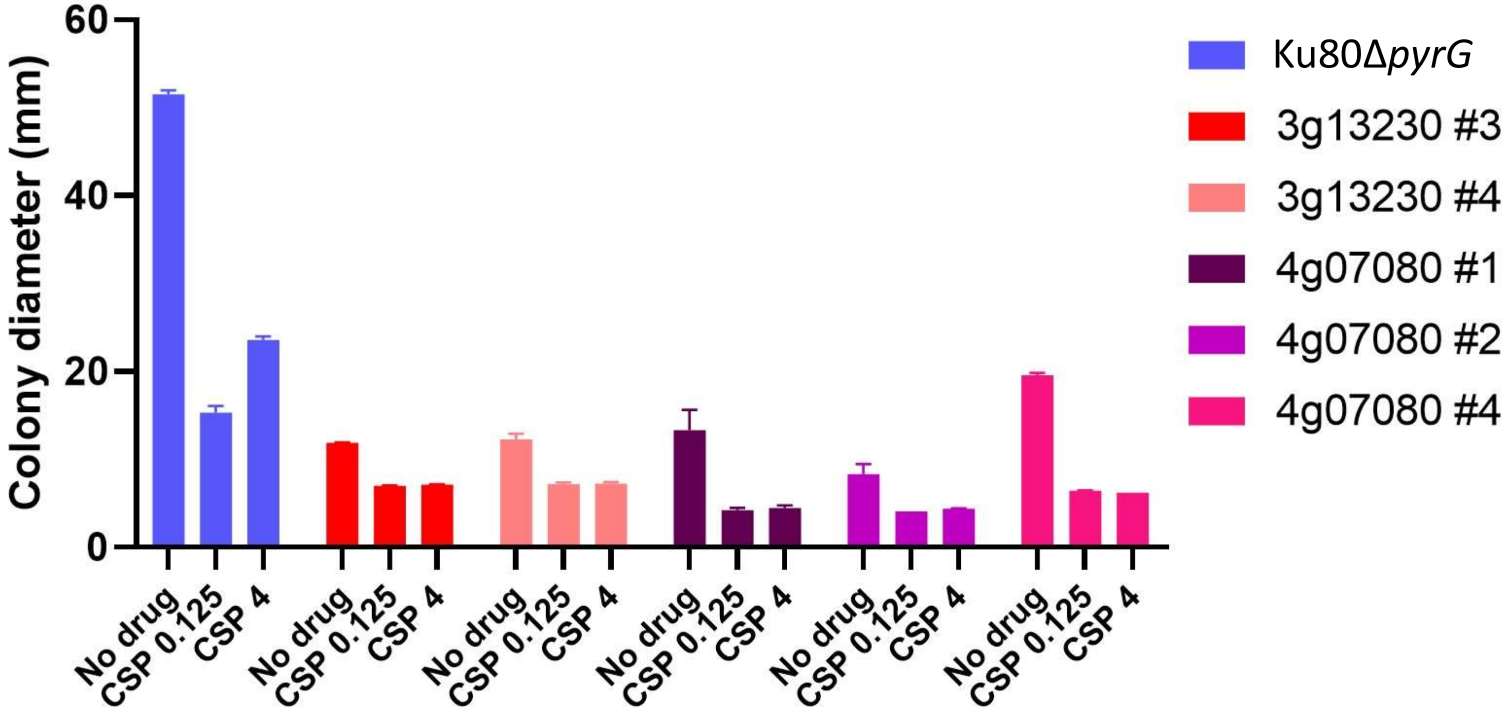

Figure S6. Loss of CPE in ΔAfu3g13230 and ΔAfu4g07080 (Δ*dscP*). Ten thousand conidia were inoculated in the center of GMM agar plates with 0, 0.125 or 4 μg/ml of caspofungin, and incubated at 37°C for 72 hours. Colony diameters were measured after 72 hours. Experiments were performed with three biological replicates per condition.

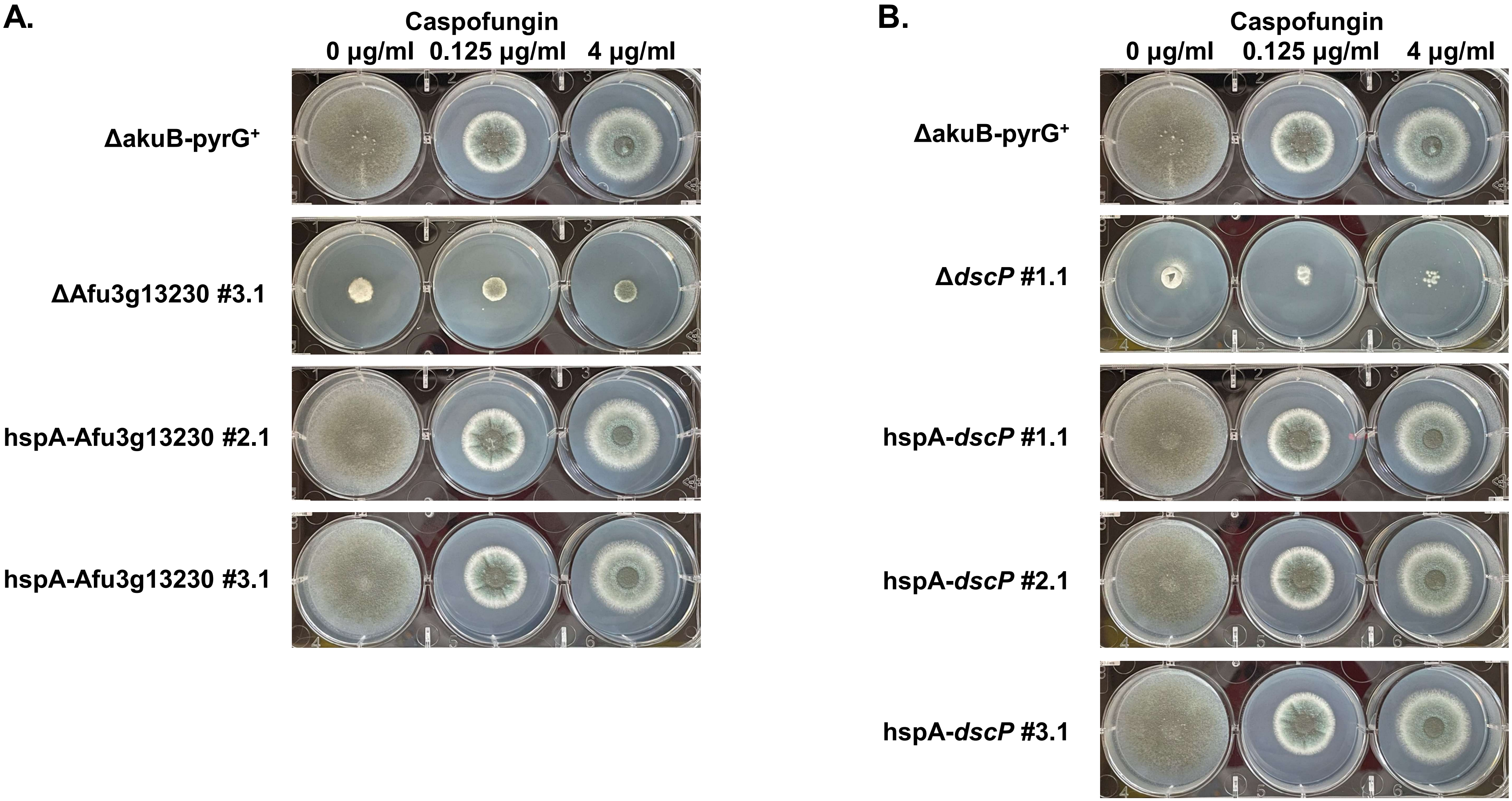

Figure S7. Growth of Afu3g13230 (A) and *dscP* (B) wild-type and mutants. Ten thousand conidia were inoculated in the center of GMM agar plates with 0, 0.125 or 4 µg/ml of caspofungin, and incubated at 37°C for 72 hours. ΔakuB-pyrG^{+} = the wild-type parental strain, ΔAfu3g13230 and Δ*dscP* = gene deletion mutants, and hspA-X = overexpression mutants. The number after each mutant represents the independent transformant.

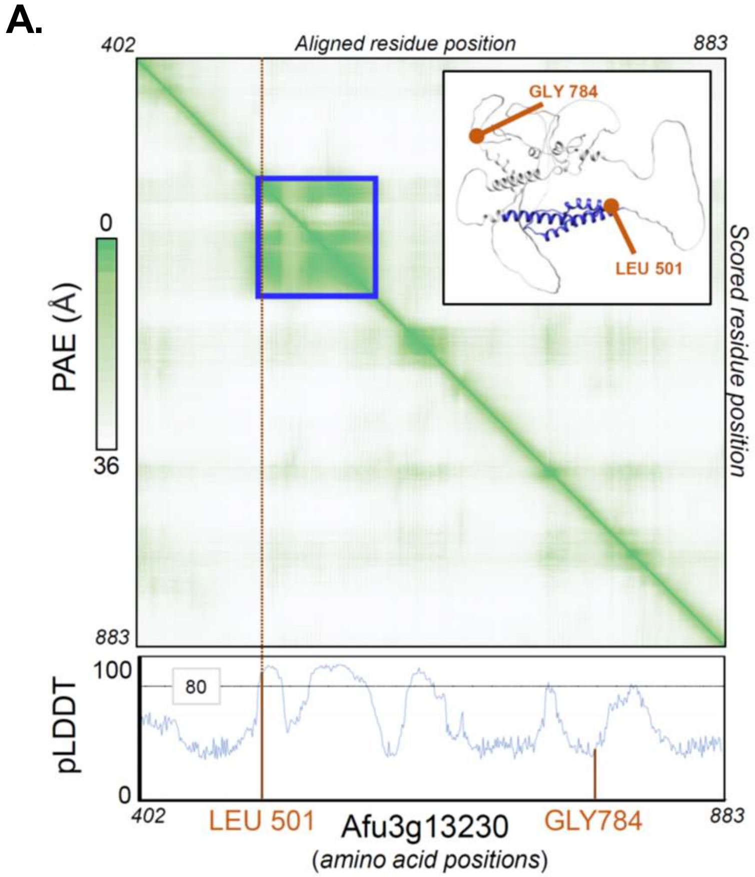

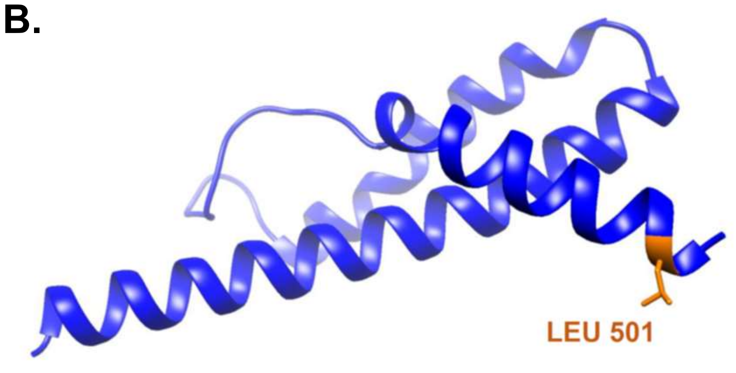

C.

Structure-Based Molecular Function - GO Term Predictions

Name	Go Term	Score
nuclease activity	GO:0004518	0.86
deoxyribonuclease activity	GO:0004536	0.85
hydrolase activity, acting on ester bonds	GO:0016788	0.83
exodeoxyribonuclease activity	GO:0016895	0.73
exodeoxyribonuclease activity	GO:0004529	0.73
nucleic acid binding	GO:0003676	0.72
organic cyclic compound binding	GO:0097159	0.7
heterocyclic compound binding	GO:1901363	0.69
exonuclease activity	GO:0016796	0.65
hydrolase activity	GO:0016787	0.59
exonuclease activity	GO:0004527	0.58
catalytic activity, acting on DNA	GO:0140097	0.57

Figure S8. AlphaFold2 model of 483 residue portion of Afu3g13230 containing two variants associated with CPE. (A) AlphaFold2 metrics for protein model. Heatmap of predicted aligned error (PAE) for all residue pairs reveals a closely packed domain (blue box) containing Leu501. Bottom: The per-residue confidence score (pLDDT) for each residue reveals that the domain containing Leu501 comprises high confidence residues (pLDDT>80). Inset: The AlphaFold2 model of Afu3g13230 with the domain containing Leu501 highlighted in blue. (B) Detail of the structural domain identified in panel A. The position of Leu501 is highlighted with the sidechain is shown. (C) Functional classification of the identified domain by DeepFRI along with the identified GO terms and scores.

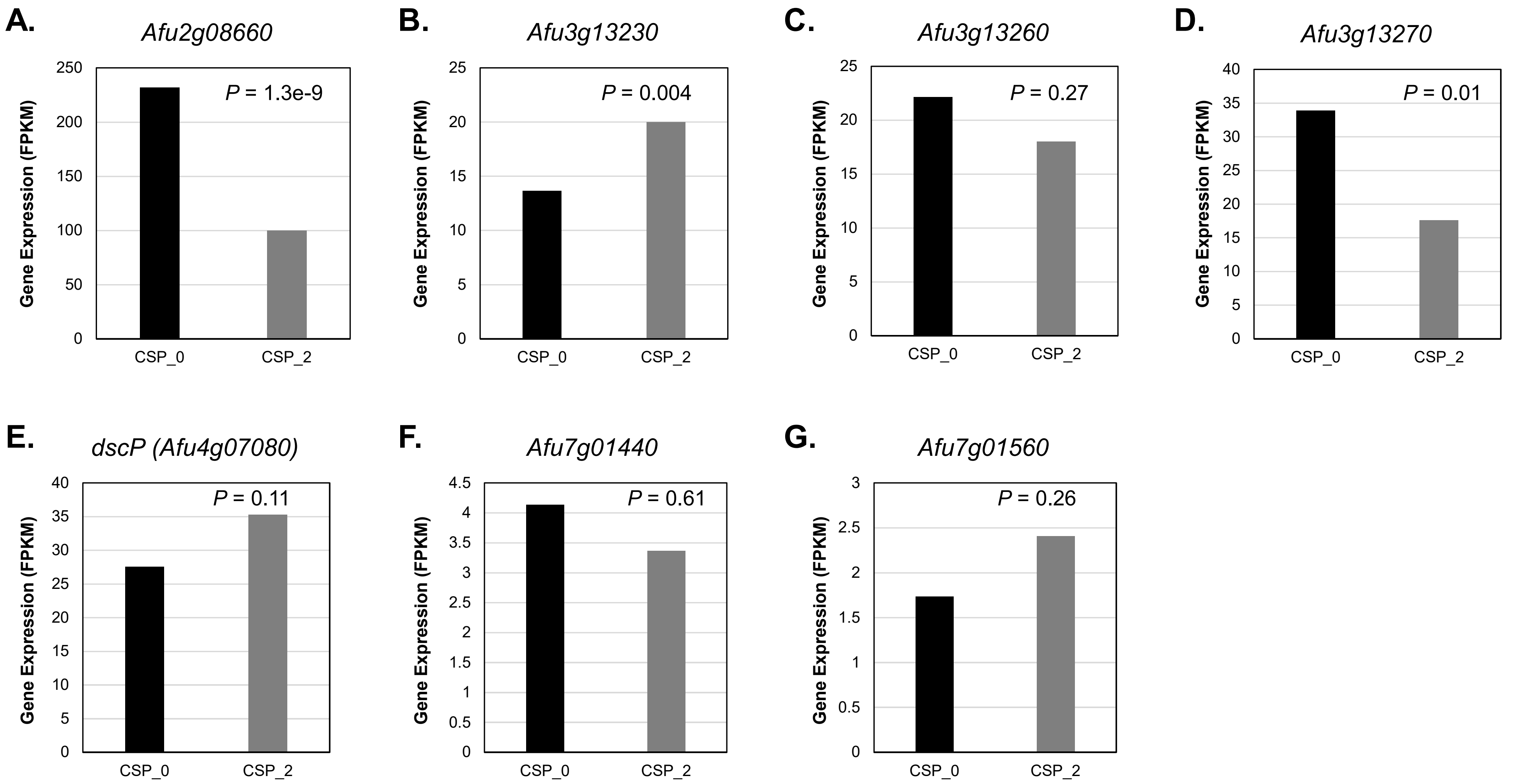

Figure S9. Expression (FPKM) in *A. fumigatus* CEA17 on minimal media with 0 ug/ml caspofungin (black bar) and 2 ug/ml caspofungin (gray bar) for genes associated with CPE and experimentally knocked out. RNA-seq data was obtained from Valero *et al.* 2020. CSP_0 = 0 μM caspofungin and CSP_2 = 2 μM caspofungin.
